# Molecular Detection of Wesselsbron Virus in Dromedary Camels, Borana Zone, Ethiopia, 2024

**DOI:** 10.3201/eid3106.250130

**Published:** 2025-06

**Authors:** Hassan Ishag, El Tigani El Tigani-Asil, Wubishet Zawde, Abdelmalik I. Khalafalla, Mohammed Albreiki, Noura Al Zarooni, Abde Mohammed, Hagos Asgedom, Getnet Abei, Tesfaye Riufael, Golo Dabasa, Derara Birasa, Jarso Debano, Ghada Abdelwahab, Shameem Habeeba, Mokonin Belexe, Gerade Abduljami, Kassaw Amssalu, Mohd Farouk Yusof, Fikru Ragassa, Asma Mohamed

**Affiliations:** Abu Dhabi Agriculture and Food Safety Authority, Abu Dhabi, United Arab Emirates (H. Ishag, E.T. El Tigani-Asil, A.I. Khalafalla, M. Albreiki, N. Al Zarooni, G. Abdelwahab, S. Habeeba, M.F. Yusof, A. Mohamed); Ministry of Agriculture, Addis Ababa, Ethiopia (W. Zawde, D. Birasa, K. Amssalu, F. Ragassa); Animal Health Institute, Sebeta, Ethiopia (A. Mohammed, H. Asgedom, G. Abei, T. Riufael); Yabello Regional Veterinary, Yabello, Ethiopia (G. Dabasa, J. Debano, M. Belexe, G. Abduljami)

**Keywords:** Wesselsbron virus, viruses, zoonoses, sick camel, Ethiopia

## Abstract

We used PCR, Sanger sequencing, and phylogenetic analysis to identify Wesselsbron virus (WSLV) clade 1 in sick camels from Borana Zone, Ethiopia. Although WSLV primarily infects sheep and cattle, its pathogenicity in camels remains unclear. Camel farmers in the region should be aware of WSLV and its health effects in camels.

Camel husbandry is crucial for the pastoral communities in East Africa; however, rising death rates among camels remain poorly understood. During 1995–2004, various pathogens, including *Mannheimia hemolytica* (*1*), morbillivirus (*2*), and *Streptococcus equi* (*3*), were associated with epidemics in camels. Since 2005, unexplained camel deaths were reported in Ethiopia, and later in Somalia and Kenya, and death rates reached 6.6% (*4*). A new disease outbreak in 2020–2021 further threatened camels in Kenya, Ethiopia, and Somalia (*5*).

On May 7, 2024, outbreaks of an unidentified camel disease were first reported in the Dubluk district of the Borana Zone of Ethiopia. The outbreak continued until July and affected 8 districts: Gomole, Arero, Dubluk, Miyo, Yabello, Dilo, Wacile, and Dhas (Appendix Figure 1). A total of 147 camels died, a case fatality rate of 70% (147/209).

In July 2024, a joint investigation team from the World Organisation for Animal Health Collaborating Centre for Camel Diseases at the Abu Dhabi Agriculture and Food Safety Authority in the United Arab Emirates and the Animal Health Institute (AHI) in Ethiopia investigated camel deaths in the Arero and Gomole districts (Appendix Figure 1). The AHI Animal Research Scientific and Ethics Review Committee granted ethics approval (approval no. ARSERC APP/002/2025). During the investigation, we examined 24 camels (16 sick and 8 recovered) <3 years of age. We observed clinical signs such as lethargy, dullness, shivering, and bilateral lacrimation that resulted in vision impairment. Other signs involved labored breathing, yawning, and nervous signs such as tremors that often progressed to recumbency (Figure 1, panel A). Some camels experienced oliguria, constipation, and death within an average of 2–3 days.

**Figure 1:**
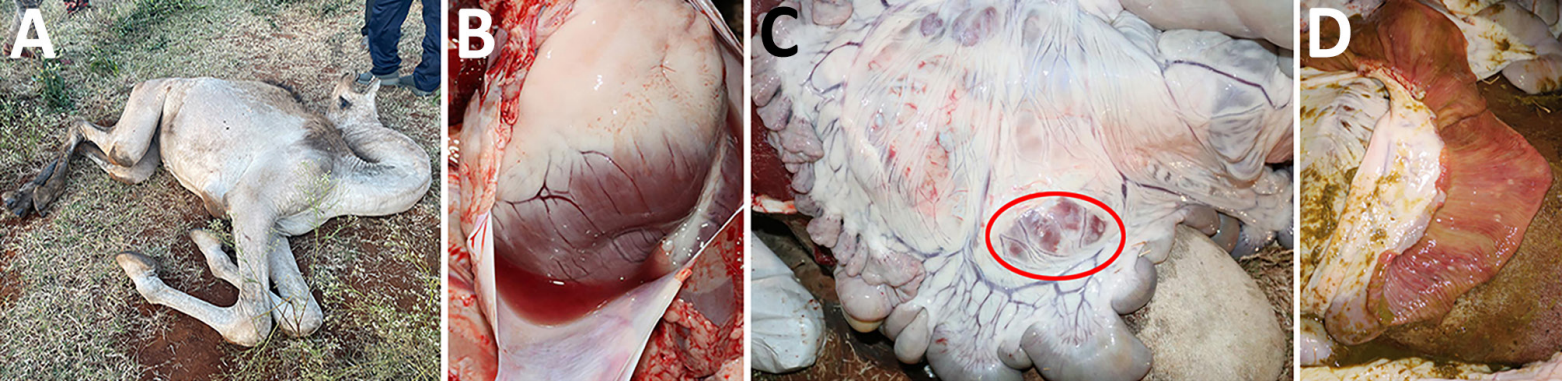
Images of clinical signs of Wesselsbron virus in dromedary camels, Borana Zone, Ethiopia, 2024. A) Recumbency in a calf that died. B–D) Necropsy images of from 2 other camels that died: B) pericardium demonstrating fluid around to the heart (hydropericardium); C) enlarged and congested myenteric lymph nodes (circled in red) in the abdomen; and D) intestine showing edema and congested mucosa.

We collected 34 nasal, oral, or ocular swab samples and 16 blood samples from sick and recovered camels. We performed necropsies on 2 euthanized camels and 1 recently dead calf. We took tissue and fluid samples (n = 35) from various organs, including the lung, spleen, liver, heart, kidney, cerebrum, cerebellum, mesenteric lymph node, intestine, mediastinal lymph node, prescapular lymph node, and pericardial fluids. We stored all samples at −20°C and fixed some tissue samples in 10% formalin at Yabello Sub-National Veterinary Laboratory, Yabello, Ethiopia. We sent the samples through the AHI laboratory to the Abu Dhabi Agriculture and Food Safety Authority laboratory. The main postmortem findings included hydropericardium (Figure 1, panel B), myenteric lymph node enlargement with congestion (Figure 1, panel C), and intestinal edema with congested mucosa (Figure 1, panel D).

A bacteriologic analysis of 19 swab samples and tissue specimens identified *Escherichia coli*, *Enterococcus* spp., *Staphylococcus* spp., *Rothia* spp., *Bacillus cereus*, *Moellerella wisconsensis*, *Corynebacterium* spp., and *Curtobacterium citreum*, which were likely environmental contaminants. Parasitologic examination of 13 fecal samples detected *Eimeria cameli* oocysts, Trichostrongylidae ova, and *Trichuris* ova.

We used genus-wide panvirus PCR assays to screen for several viruses, including bluetongue virus, bovine viral diarrhea virus, coronaviruses, peste des petits ruminants virus, paramyxovirinae, parapoxviruses, and orthoflavivirus. We also conducted targeted assays for Rift Valley fever, foot and mouth disease, enzootic bovine leukosis, Crimean Congo hemorrhagic fever, and camelpox viruses (Appendix Table).

All assays returned negative results, except for the panflavivirus reverse transcription PCR targeting the *NS5* gene (*6*), which detected orthoflavivirus in 18 (51.4%) of 35 necropsy samples and 3 (8.8%) of 34 swab samples (Appendix Figure 2); we found no positive results in blood samples. A Wesselsbron virus (WSLV)–specific real-time PCR (*7*) considerably improved detection, identifying WSLV in 25 (71.4%) of 35 tissue samples, and 3 (18.8%) of 16 blood samples, whereas swab samples had the same results as the panflavivirus assay. Detection of the virus in all 3 necropsied camels and multiple tissue samples suggests viremia and systemic infection.

We selected 15 (83.3%) of 18 panflavivirus-positive tissue samples for Sanger sequencing on the basis of the quality of the PCR bands. The National Center for Biotechnology Information BLAST (https://blast.ncbi.nlm.nih.gov) analysis showed that the sequences had 97%–98% identity with existing WSLV sequences. We deposited the sequences in GenBank (accession nos. PQ672109–PQ672123). The sequences aligned with National Center for Biotechnology Information data and previously published WSLV sequences. A phylogenetic analysis using the maximum-likelihood method indicated sequences were in WSLV clade 1 (Figure 2).

**Figure 2:**
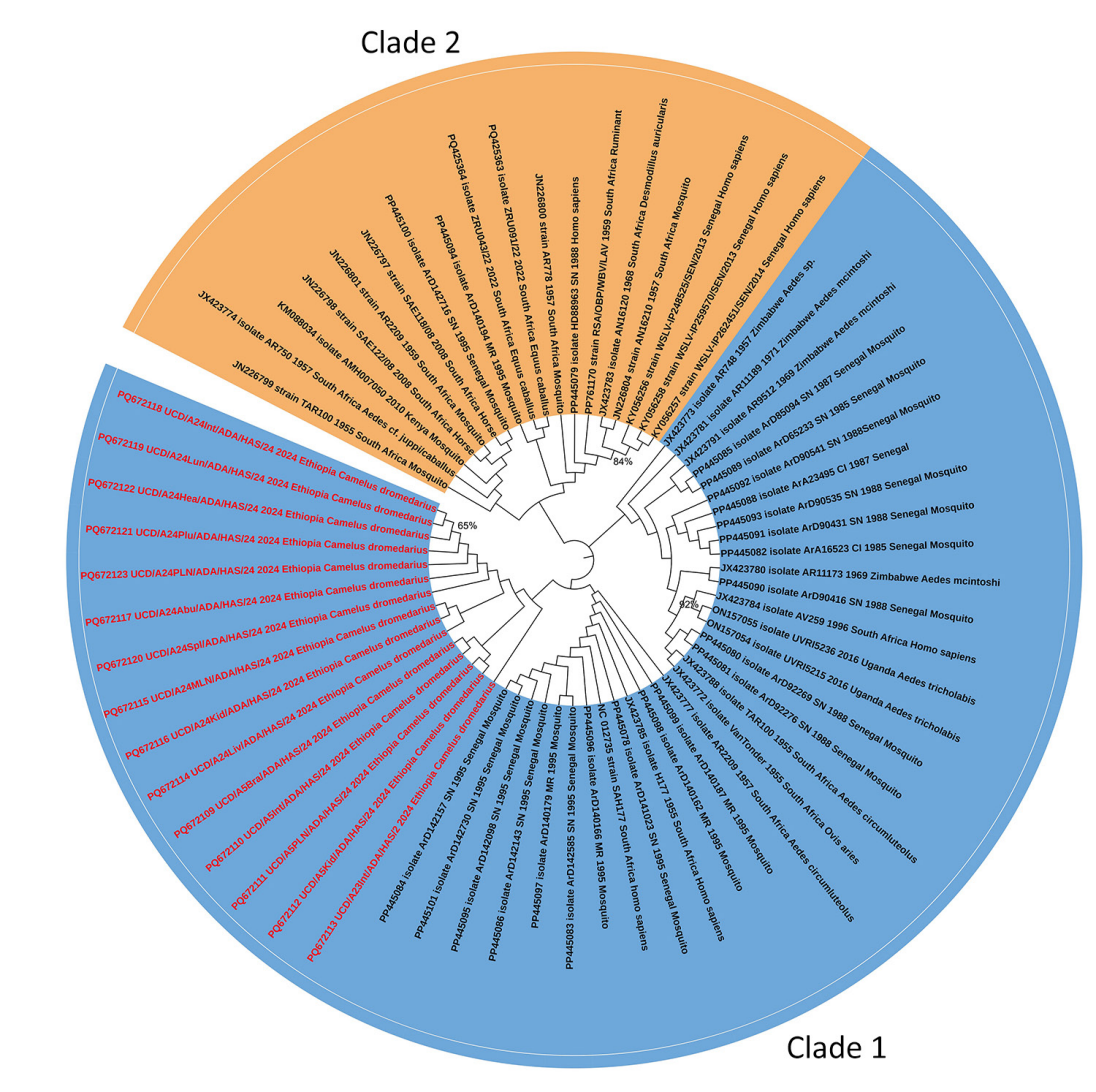
Phylogenetic analysis of Wesselsbron virus (WSLV) in dromedary camels, Borana Zone, Ethiopia, 2024. The maximum-likelihood phylogenetic tree was constructed with 1,000 bootstrap replicates based on 65 partial *NS5* gene sequences of WSLV, including the camel-derived WSLV sequences (maximum length 252 bp). The newly identified camel WSLV strains from this study are in red within clade 1.

This study comprehensively analyzed WSLV in various tissue, swab, and blood samples from affected camels. The identified virus strain belongs to clade 1, which is recognized for its pathogenicity and neurotropism (*8*). WSLV is associated with Wesselsbron disease, which can cause reproductive, neurologic, and systemic effects in various hosts, including humans, livestock, and rodents (*9*,*10*).

In conclusion, we identified WSLV in sick camels, and provided a WSLV partial gene sequence derived from camels. Whole-genome sequencing, virus isolation, and experimental infection testing in healthy camels are needed to understand the pathogenicity and to address existing knowledge gaps of this virus. Nonetheless, this detection expanded the known geographic range of WSLV to Ethiopia and farmers should be aware of this virus and its effects in camels. 

Appendix Additional information for molecular detection of Wesselsbron virus in dromedary camels, Borana Zone, Ethiopia, 2024
