# Supplementary material for: Molecular Detection of Wesselsbron Virus in Dromedary Camels, Borana Zone, Ethiopia, 2024
Source: Emerg Infect Dis. 2025 Jun;31(6):1263–5. doi: 10.3201/eid3106.250130 (PMC12123917; doi:10.3201/eid3106.250130)
Supplement: Appendix — Additional information for molecular detection of Wesselsbron virus in dromedary camels, Borana Zone, Ethiopia, 2024 [file 25-0130-Techapp-s1.pdf]

Article DOI: https://doi.org/10.3201/eid3106.250130

EID cannot ensure accessibility for Supplemental Materials supplied by authors. Readers who have difficulty accessing supplementary content should contact the authors for assistance.

Molecular Detection of Wesselsbron Virus in Dromedary Camels, Borana Zone, Ethiopia, 2024

Appendix

References

1. Mulholland C, McMenamy MJ, Hoffmann B, Earley B, Markey B, Cassidy J, et al. The development of a real-time reverse transcription-polymerase chain reaction (rRT-PCR) assay using TaqMan technology for the pan detection of bluetongue virus (BTV). J Virol Methods. 2017;245:35–9. PubMed https://doi.org/10.1016/j.jviromet.2017.03.009

2. Hoffmann B, Depner K, Schirrmeier H, Beer M. A universal heterologous internal control system for duplex real-time RT-PCR assays used in a detection system for pestiviruses. J Virol Methods. 2006;136:200–9. PubMed https://doi.org/10.1016/j.jviromet.2006.05.020

3. Watanabe S, Masangkay JS, Nagata N, Morikawa S, Mizutani T, Fukushi S, et al. Bat coronaviruses and experimental infection of bats, the Philippines. Emerg Infect Dis. 2010;16:1217–23. PubMed https://doi.org/10.3201/eid1608.100208

4. Flannery J, Rajko-Nenow P, Arnold H, Weezep E, van Rijn PA, Ngeleja C, et al. Improved PCR diagnostics using up-to-date in silico validation: an F-gene RT-qPCR assay for the detection of all four lineages of peste des petits ruminants virus. J Virol Methods 2019;274:113735. PubMed https://doi.org/10.1016/j.jviromet.2019.113735.

5. Tong S, Chern SW, Li Y, Pallansch MA, Anderson LJ. Sensitive and broadly reactive reverse transcription-PCR assays to detect novel paramyxoviruses. J Clin Microbiol. 2008;46:2652–8. PubMed https://doi.org/10.1128/JCM.00192-08

6. Moureau G, Temmam S, Gonzalez JP, Charrel RN, Grard G, de Lamballerie X. A real-time RT-PCR method for the universal detection and identification of flaviviruses. Vector Borne Zoonotic Dis. 2007;7:467–77. PubMed https://doi.org/10.1089/vbz.2007.0206

7. Drosten C, Göttig S, Schilling S, Asper M, Panning M, Schmitz H, et al. Rapid detection and quantification of RNA of Ebola and Marburg viruses, Lassa virus, Crimean-Congo hemorrhagic fever virus, Rift Valley fever virus, dengue virus, and yellow fever virus by real-time reverse transcription-PCR. J Clin Microbiol. 2002;40:2323–30. PubMed https://doi.org/10.1128/JCM.40.7.2323-2330.2002

8. Callahan JD, Brown F, Osorio FA, Sur JH, Kramer E, Long GW, et al. Use of a portable real-time reverse transcriptase-polymerase chain reaction assay for rapid detection of foot-and-mouth disease virus. J Am Vet Med Assoc. 2002;220:1636–42. PubMed https://doi.org/10.2460/javma.2002.220.1636

9. Rola-Łuszczak M, Finnegan C, Olech M, Choudhury B, Kuźmak J. Development of an improved real time PCR for the detection of bovine leukaemia provirus nucleic acid and its use in the clarification of inconclusive serological test results. J Virol Methods. 2013;189:258–64. PubMed https://doi.org/10.1016/j.jviromet.2013.02.014

10. Wölfel R, Paweska JT, Petersen N, Grobbelaar AA, Leman PA, Hewson R, et al. Virus detection and monitoring of viral load in Crimean-Congo hemorrhagic fever virus patients. Emerg Infect Dis. 2007;13:1097–100. PubMed https://doi.org/10.3201/eid1307.070068

11. Zhao H, Wilkins K, Damon IK, Li Y. Specific qPCR assays for the detection of orf virus, pseudocowpox virus and bovine papular stomatitis virus. J Virol Methods. 2013;194:229–34. PubMed https://doi.org/10.1016/j.jviromet.2013.08.027

Appendix Table. List of primers and probe sequences along with the references for the real-time PCR assays applied during the investigation of the cause of camel death in Boran Zone, Ethiopia*

Test	Primer sequences (5′-3′)	Reference
Pan-bluetongue virus assay	BTV Seg-10_F: AARGCGGAGAARGCTGCAT<br>BTV Seg-10_FR: ARYCTGACRTCATCACGAAACG<br>BTV Seg-10_P: FAM-CGCATCGTACGCRGAA-MGB	(*1*)
Pan-bovine viral diarrhea virus assay	BVD 190-F: GRAGTCGTCARTGGTTCGAC<br>V326: TCAACTCCATGTGCCATGTAC<br>TQ-pesti: FAM-TGCYAYGTGGACGAGGGCATGC-TAMRA	(*2*)
Pan-coronaviruses assay	Round 1:<br>CoV-RVS2: GGTTGGGAYTAYCCHAARTGTGA<br>CoV-FWD3: CCATCATCASWYRAATCATCATA<br>Round 2:<br>CoV-FWD4/Other: GAYTAYCCHAARTGTGAUMGWGC<br>Reverse: Same as round 1 (CoV-FWD3)	(*3*)
Pan-peste des petits ruminants assay	PPRFF: CATAGSACTGGCAGCTTGCA<br>PPRFR: GAGCCCTGGGTTGATTTTRG<br>PPRFProbe: FAM-CTTGTCACATTAATATGCTG-BHQ1	(*4*)
Pan-paramyxovirinae	Round 1:<br>PAR-F1: GAAGGITATTGTCAIAARNTNTGGAC<br>PAR-R: GCTGAAGTTACIGGITCICCDATRTTNC<br>Round 2:<br>PAR-F2: GTTGCTTCAATGGTTCARGGNGAYAA<br>Reverse: Same as round-1 reverse primer (PAR-R)	(*5*)
Panflavivirus assay	Flavi-FWD: TGYRTBTAYAACATGATGGG<br>Flavi-RVS: GTGTCCCAICCNGCNGTRTC	(*6*)
Rift valley fever	RVS: AAAGGAACAATGGACTCTGGTCA<br>RVAs: CACTTCTTACTACCATGTCCTCCAAT<br>RVP: FAM-AAAGCTTTGATATCTCTCAGTGCCCCAA-BHQ1	(*7*)
Foot-and-mouth disease	Callahan 3DF: ACTGGGTTTTACAAACCTGTGA<br>Callahan 3DR: GCGAGTCCTGCCACGGA<br>Callahan 3D: FAM-TCCTTTGCACGCCGTGGGAC-TAMRA	(*8*)
Enzootic bovine leukosis	MRBLVL: CCTCAATTCCCTTTAAACTA<br>MRBLVR: GTACCGGGAAGACTGGATTA<br>MRBLV probe: FAM-GAACGCCTCCAGGCCCTTCA BHQ1	(*9*)
Crimean-Congo hemorrhagic fever	CCHFV-F: CAAGGGGTACCAAGAAAATGAAGAAGGC<br>CCHFV-R: GCCACAGGGATTGTTCCAAAGCAGAC<br>CCHFV-Probe: FAM-ATCTACATGCACCCTGCTGTGTTGACA-BHQ1<br>SE03: FAM-ATTTACATGCACCCTGCCGTGCTTACA-BHQ1<br>SE0A: FAM-AGCTTCTTCCCCCACTTCATTGGAGT-BHQ1	(*10*)
Camelpox virus	CMLV-F: GATGCGGATCTTTATGATAC<br>CMLV-R: GCTGTAATACCAAATACTTCA<br>CMLV-Probe: FAM-ACCATCTACTGTATCACCAACAACTGT-BHQ-1	In-house
Pan-parapox viruses	Forward: CGCGGTCTGGTCCTTG<br>Reverse: CAGCATCAACCTCTCCTACATCA<br>Probe: Fam-CCACGAAGCTGCGCAGCAT-BHQ1	(*11*)

*CCHFV, Crimean-Congo hemorrhagic fever virus; F, forward; R, reverse.

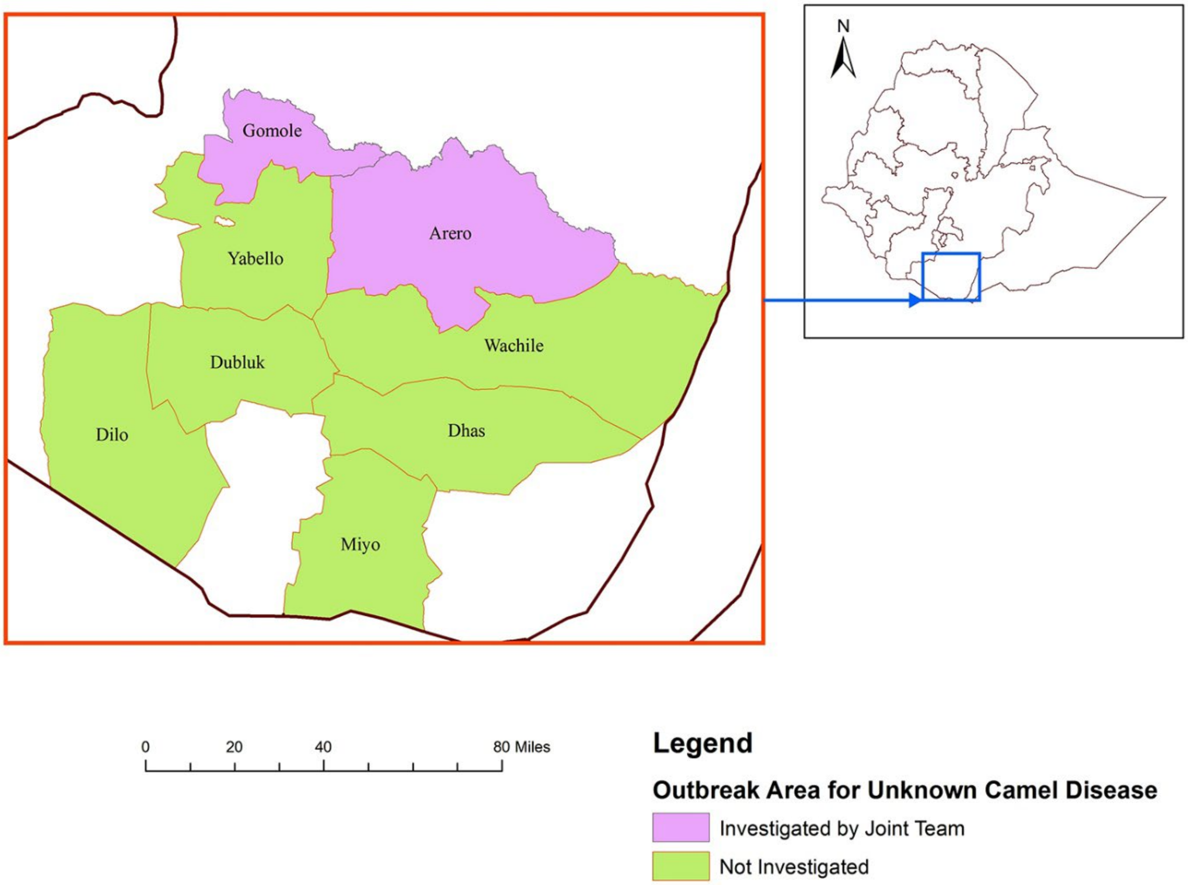

Appendix Figure 1. Camel outbreak areas investigated by the ADAFSA and AHI Joint team.

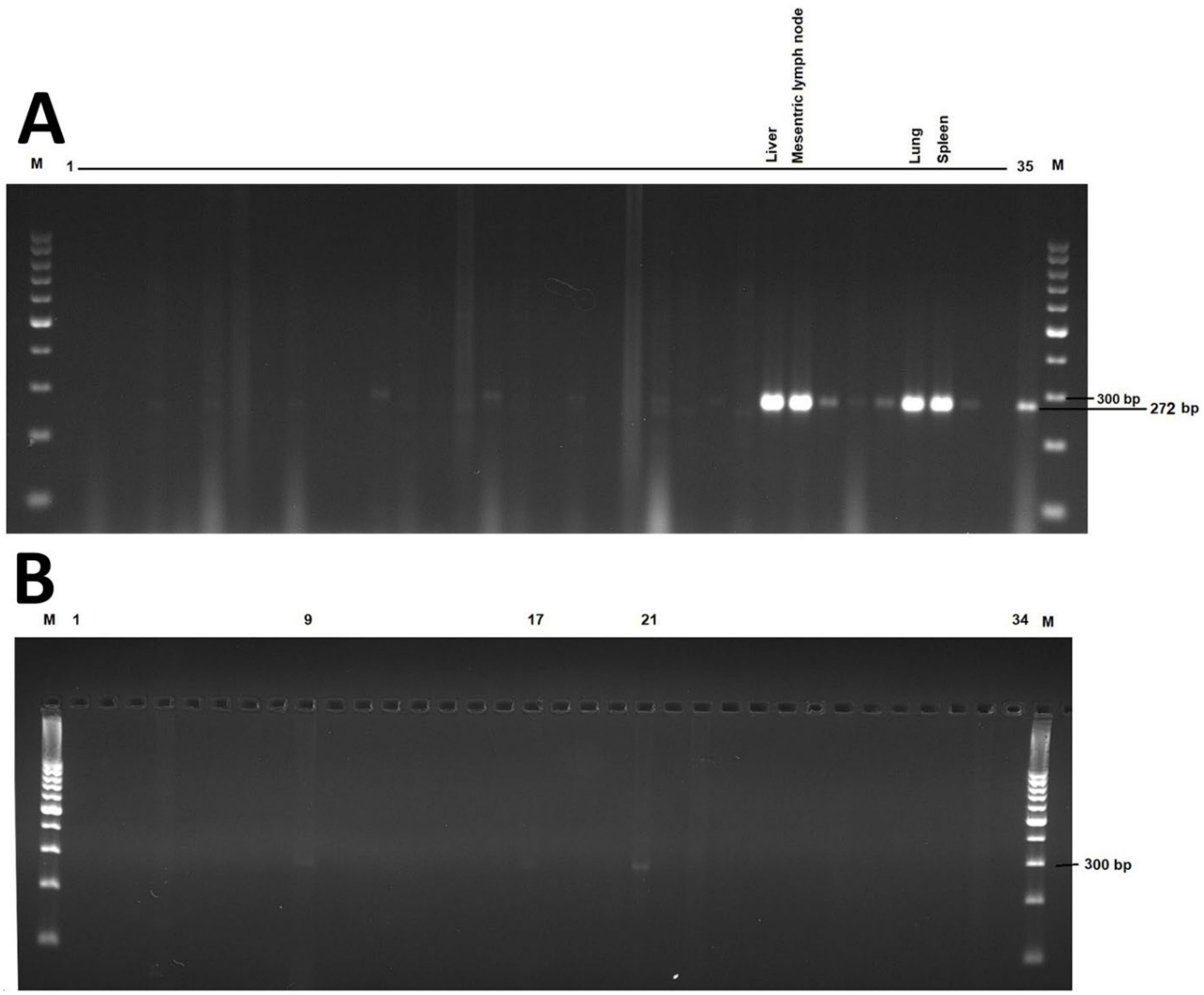

Appendix Figure 2. Gel-based panflavivirus RT-PCR assay. Samples collected from necropsied animals (1–35) (A), and swabs from sick animals (1–34) (B) are shown, with the characteristic band size of the panflavivirus RT-PCR assay (272 bp) indicated. Samples with prominent bands are indicated.
